# Digital engagement and physical activity patterns across Chinese populations: a secondary analysis of national survey data

**DOI:** 10.3389/fpubh.2026.1726172

**Published:** 2026-05-20

**Authors:** Yuxin Zhou, Liwen Xu

**Affiliations:** 1 College of Physical Education, Jingdezhen Ceramic University, Jingdezhen, China; 2 Department of Basic Courses, Jiangxi Aviation Vocational and Technical College, Nanchang, China

**Keywords:** China, digital divide, digital health, health disparities, physical activity

## Abstract

**Background:**

The relationship between digital engagement and physical activity among Chinese populations remains unclear. This study examined this association and identified population-specific effects.

**Methods:**

We analyzed data from 33,613 participants from CFPS 2020 and CHARLS 2020. Digital engagement was measured using a composite index (0–100) covering digital access, skills, health information behavior, and service use. Physical activity was assessed using IPAQ standards (MET-minutes/week). Multilevel mixed-effects models examined associations, with moderation analyses for age, urban–rural, and educational differences.

**Results:**

Mean digital engagement was 48.3 ± 22.1, with marked urban–rural disparities (61.2 vs. 32.5, *p* < 0.001). Each 10-point increase in digital engagement was associated with 260 MET-minutes/week increase in physical activity (95% CI: 220–300, *p* < 0.001). The association showed an inverted U-shaped age pattern, strongest among 45–59 years (*β* = 0.42). Despite lower digital engagement, rural areas showed stronger effects (*β* = 0.34) than urban (*β* = 0.23, p-interaction = 0.008). Health information searching (*β* = 0.38) and digital health service use (*β* = 0.35) had stronger effects than digital access alone (*β* = 0.21).

**Conclusion:**

Digital engagement positively associates with physical activity, particularly benefiting middle-aged adults and rural residents. Targeted digital health interventions could effectively promote physical activity in underserved populations.

## 1 Introduction

Digital transformation has become a pivotal driver of global health promotion initiatives (1). China, as one of the world’s largest digital economies, has 1.08 billion internet users, representing a penetration rate of 75.6%. Among these users, 99.8% access the internet through mobile devices (CNNIC, 2024). This unprecedented digital penetration offers opportunities for health behavior interventions, yet raises concerns about digital divides potentially exacerbating health inequities (2). Physical inactivity, the fourth leading risk factor for global mortality, accounts for 3.2 million deaths annually (57). In China, although universal fitness has been elevated to a national strategy under the “Healthy China 2030” initiative, national physical fitness monitoring reveals that only 23.8% of urban residents and 15.3% of rural residents meet the national recommended exercise standards (General Administration of Sport of China, 2020) (3). These national standards, defined by China’s General Administration of Sport, require adults to engage in moderate-intensity physical activity for at least 150 min per week or vigorous-intensity activity for at least 75 min per week—criteria that align with the World Health Organization (WHO) 2020 guidelines on physical activity and sedentary behavior (57). This alignment facilitates international comparisons of our findings. Concurrently, fitness applications have attracted 230 million monthly active users (58). However, the actual effectiveness of digital tools in promoting physical activity and their differential impacts across populations remain unclear, particularly given China’s vast geographic expanse and uneven development patterns (4).

This study integrates three complementary theoretical perspectives. First, the three-tier digital divide model—encompassing access, skills, and outcome disparities—informed our four-dimensional digital engagement index, reflecting the progression from mere access to meaningful health utilization (5). Second, the social-ecological model guided our adoption of three-level mixed-effects models (individuals within communities within provinces) and cross-level interactions (6, 7). Third, behavior change theory guided our mechanism interpretation, helping explain why active health information seeking demonstrated stronger associations than passive digital access (8, 9). Importantly, these frameworks must be situated within an understanding of physical activity domain heterogeneity. Total physical activity, as measured by MET-minutes, comprises occupational, transportation, and leisure-time domains that differ substantially between populations. In China, rural residents typically accumulate higher total MET-minutes than their urban counterparts, largely due to agricultural labor rather than volitional exercise. Because digital engagement is theorized to promote health-conscious, self-directed physical activity rather than occupational exertion, we distinguish between total physical activity and leisure-time physical activity (LTPA) throughout our analyses. This domain-specific approach ensures that observed associations reflect the influence of digital engagement on volitional exercise behavior, providing a more theoretically coherent test of the hypothesized mechanisms. These frameworks collectively guided variable operationalization, model specification, and result interpretation.

International evidence demonstrates the positive impact of digital health interventions on physical activity. A cross-national European study involving 17 countries found that digital health literacy was positively associated with physical activity (OR = 1.42), while mobile app interventions increased physical activity by 12–20% (10), and wearable device users increased their daily steps by 2,000 (11, 12). Asian research revealed that health app use among Japanese older adults was associated with a 25% increase in physical activity, and digital health service users in South Korea demonstrated 40% higher exercise adherence (13–15). However, these findings predominantly originate from high-income countries, and their applicability to the Chinese context remains questionable.

Research in the Chinese context remains limited and exhibits several methodological limitations. A small-sample Beijing study found that exercise app users exercised 45 min more per week (16), while a Shanghai survey showed that only only 12% of older adults used digital health tools, though users’ achievement rate was 2.3 times that of non-users (17). Existing studies are constrained by limited sample representativeness, inadequate control for selection bias, lack of urban–rural comparisons (18), and particularly fail to systematically explore the differential impacts of various dimensions of digital engagement and the role of moderating factors.

Given this context, this study utilizes nationally representative data to systematically analyze the relationship between digital engagement and physical activity among different Chinese populations. The study addresses the following research questions: (1) What is the association between digital engagement and physical activity? (2) How does this association differ across populations? (3) How does the urban–rural digital divide affect physical activity patterns? (4) Which components of digital engagement demonstrate the strongest effects? Based on theoretical frameworks and existing evidence, we propose the following hypotheses: H1: Higher digital engagement is positively associated with increased physical activity levels; H2: Association strength varies across age groups, with the strongest effects among middle-aged adults; H3: Urban–rural disparities exist in both digital engagement and physical activity patterns; H4: Education and income moderate the relationship between digital engagement and physical activity. The significance of this study lies in its provision of the first nationally representative empirical evidence from China, identification of priority intervention populations, provision of scientific basis for the Healthy China 2030 initiative, and insights for developing countries facing similar challenges.

## 2 Methods

### 2.1 Study design

**Figure 1:**
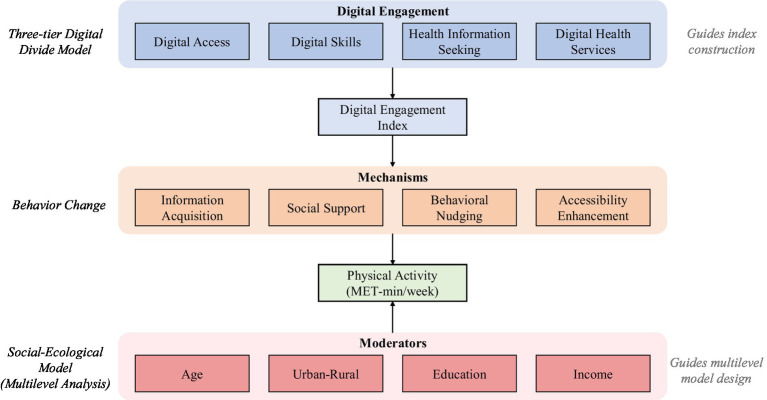
Conceptual framework of digital engagement on physical activity.

This study conducted a secondary analysis of cross-sectional survey data, employing a multilevel analytical framework that integrates multiple nationally representative data sources to explore the relationship between digital engagement and physical activity (as illustrated in Figure 1). The study adheres to the STROBE statement for observational study reporting standards to ensure methodological transparency and reproducibility.

### 2.2 Data sources

Data primarily derive from two large-scale national surveys. The China Family Panel Studies (CFPS) 2020 data provided the main analytical sample. This survey employed multistage, implicit stratification, probability sampling proportional to population size, covering 25 provinces and representing 95% of China’s population. The remaining 5% not covered by CFPS includes residents of Tibet, Qinghai, Xinjiang, Ningxia, Inner Mongolia, Hainan, and Hong Kong/Macao/Taiwan. These regions were excluded primarily due to logistical challenges (remote geography, low population density) and administrative considerations (special administrative regions). Notably, these excluded regions are characterized by lower population density, higher proportions of ethnic minorities, and in some cases (Tibet, Qinghai, Xinjiang) lower internet penetration rates and distinct physical activity patterns (e.g., pastoralist lifestyles). The 2020 survey successfully interviewed 14,218 individuals from 4,932 households, with a response rate of 82.3%. CFPS data contains over 500 variables covering digital device use, internet behavior, physical exercise, health status, and detailed sociodemographic information.

Both surveys employed Computer-Assisted Personal Interviewing (CAPI) as the primary method. Due to COVID-19, approximately 15–20% of 2020 interviews used telephone interviewing (CATI); interview mode was included in sensitivity analyses. Regarding the potential influence of the COVID-19 pandemic on study variables, several considerations are noteworthy. First, CFPS 2020 fieldwork was conducted primarily between July and December 2020, after the most stringent nationwide lockdowns (January–April 2020) had been lifted in most provinces. Similarly, CHARLS 2020 data collection occurred predominantly in the second half of 2020, when community-level restrictions had substantially eased across most regions. Second, we conducted sensitivity analyses controlling for interview timing (pre- versus post-September 2020, when most provinces had fully resumed normal activities) and provincial-level lockdown severity (measured by cumulative lockdown duration). These analyses yielded results consistent with our main findings (*β* = 0.25, 95% CI: 0.21–0.29 for the timing-controlled model). Third, while the pandemic undoubtedly accelerated digital adoption and altered activity patterns, this context may enhance rather than diminish the study’s relevance, as it reflects an accelerated digital health landscape that previews post-pandemic conditions in which digital tools play an increasingly central role in health behavior.

The China Health and Retirement Longitudinal Study (CHARLS) 2020 data supplemented information for those aged 45 and above, covering 28 provinces, 150 counties, and 450 villages, successfully interviewing 19,395 middle-aged and older adults individuals with a response rate of 85.7%. Additionally, the study integrated macro-level data including the 52nd Statistical Report on China’s Internet Development (CNNIC, 2024), National Physical Fitness Monitoring Bulletin (2020), provincial health literacy monitoring data (2019–2023), and provincial digital economy development indices (2023) to provide environmental variables for multilevel analysis.

To harmonize data across the two surveys, we implemented rigorous procedures: (1) using CFPS as primary source for overlapping age groups to avoid duplicate sampling; (2) standardizing variable definitions using crosswalk tables (Supplementary Table S1); (3) recalibrating survey weights with post-stratification to 2020 Census benchmarks. Sensitivity analyses using separate CFPS-only and CHARLS-only samples confirmed robustness to data pooling.

### 2.3 Variable measurement

#### 2.3.1 Dependent variable

Physical activity was measured using International Physical Activity Questionnaire (IPAQ) standards, quantified through Metabolic Equivalent Tasks (MET). Specific calculations: vigorous activities at 8.0 METs (minutes × days × 8.0), moderate-intensity activities at 4.0 METs, and walking at 3.3 METs, with the sum representing total weekly physical activity. According to WHO recommendations, physical activity was categorized as low (<600 MET-minutes/week), moderate (600–3,000 MET-minutes/week), and high (>3,000 MET-minutes/week). Achievement of WHO-recommended minimum of 150 min of moderate-intensity or 75 min of vigorous-intensity activity per week was also recorded.

We additionally disaggregated physical activity into occupational, transportation, and leisure-time domains based on IPAQ protocols. This distinction is important because rural residents’ high total activity often derives from agricultural labor rather than leisure exercise. We conducted supplementary analyses using leisure-time physical activity (LTPA) as the outcome to examine whether digital engagement specifically promotes volitional exercise behavior.

#### 2.3.2 Independent variable

Digital engagement was constructed as a 0–100 composite index through a two-stage process. First, principal component analysis (PCA) validated the hypothesized four-dimensional structure: the first four components explained 68.7% of variance, with factor loadings >0.65, KMO = 0.83, Bartlett’s test *p* < 0.001. Second, we adopted equal weighting (each dimension 25%) rather than PCA-derived weights because: (1) equal weighting enhances interpretability; (2) PCA weights are sample-specific; (3) our theoretical framework posits all dimensions as conceptually essential. Sensitivity analyses using PCA-derived weights confirmed robustness (see Sensitivity Analysis). The four dimensions comprised: (1) Digital access: internet access, smartphone/computer ownership, broadband connection; (2) Digital skills: internet use frequency, variety of digital activities, online service proficiency, digital payment use; (3) Health information behavior: online health information searching, health app use, online consultation, health information sharing; (4) Digital health services: online appointments, electronic health record access, telemedicine, health monitoring devices. Note that survey items assessed general health app use without distinguishing app types (fitness vs. medical information vs. chronic disease management), and health monitoring devices were assessed as a composite category.

#### 2.3.3 Covariates

Covariate selection was guided by the social-ecological model and informed by prior literature examining determinants of both digital engagement and physical activity. We included variables identified as potential confounders—factors associated with both the exposure (digital engagement) and outcome (physical activity)—to minimize confounding bias. Sociodemographic variables included age (as continuous and categorical variables: <18, 18–44, 45–59, ≥60 years), gender, education level (primary or below, junior high, senior high, college or above), and marital status (married, single, divorced or widowed). Age and gender were included given their documented associations with both digital engagement and physical activity patterns (19, 20). Education was included as a key determinant of digital literacy and health behaviors (1). Socioeconomic variables included per capita household income (grouped by quartiles), employment status, occupation type, and medical insurance type. Medical insurance type in China includes three main categories: Urban Employee Basic Medical Insurance (UEBMI, for formal sector employees), Urban–Rural Resident Basic Medical Insurance (URRBMI, combining the former New Rural Cooperative Medical Scheme and Urban Resident Basic Medical Insurance), and commercial/private insurance. These categories reflect both employment status and socioeconomic position, as UEBMI typically provides more comprehensive coverage. These variables were included as economic resources and occupational demands influence both digital access and physical activity opportunities (2, 21). Geographic variables included urban–rural residence, province, regional division (Eastern, Central, Western) —a standard geographic classification in China where Eastern regions (e.g., Beijing, Shanghai, Guangdong) are most economically developed with highest digital infrastructure, Central regions (e.g., Henan, Hubei) represent moderate development, and Western regions (e.g., Sichuan, Yunnan, Gansu) are less developed with lower internet penetration— and county-level economic development index. The county-level economic development index is a composite measure published annually by China’s National Bureau of Statistics, integrating indicators of GDP per capita, fiscal revenue, industrial output, and fixed asset investment at the county level (approximately 2,800 counties nationwide). This index is widely used in Chinese health research to capture local socioeconomic context, as counties represent the fundamental administrative unit for resource allocation and public service delivery in China’s governance structure—analogous to municipalities or districts in other countries. We categorized this index into quartiles, with higher quartiles indicating greater economic development. For urban–rural residence, we employed both a binary classification (urban vs. rural) for comparability with prior literature and a five-category classification capturing the urban–rural continuum: (1) urban core (central districts of prefecture-level cities), (2) urban periphery (suburban districts), (3) county towns, (4) rural townships, and (5) remote villages.

The binary urban–rural classification was determined using household registration (hukou) status, a fundamental administrative distinction in China that determines access to local public services including healthcare and education. Specifically, respondents with “agricultural hukou” were classified as rural, while those with “non-agricultural hukou” were classified as urban. For the five-category classification, we used a combination of three variables: (1) hukou status, (2) current residence location (administrative level of the community), and (3) community infrastructure characteristics (availability of paved roads, tap water, and internet connectivity as recorded in the community questionnaire).

Several ambiguous classification scenarios required explicit rules: (a) Rural-to-urban migrants (individuals with agricultural hukou living in urban areas, approximately 12.3% of our sample) were classified as “urban” in sensitivity analyses based on current residence, but as “rural” in main analyses based on hukou to maintain consistency with official Chinese statistics and prior literature; (b) Urban villages (chengzhongcun)—former rural communities now surrounded by urban development—were classified based on their administrative designation and infrastructure score, with those meeting ≥2 of 3 infrastructure criteria classified as “urban periphery”; (c) Suburban residents in rapidly urbanizing areas were classified based on the administrative level of their community (township vs. street office). We conducted sensitivity analyses using current residence instead of hukou, yielding consistent results (*β* = 0.25, 95% CI: 0.21–0.29). Sensitivity analyses examined whether the digital engagement-physical activity association varied across this gradient. Urban–rural and regional variables were included given substantial geographic disparities in both digital infrastructure and physical activity environments in China (18, 20). Health-related variables included self-rated health (5-point Likert scale), chronic disease status, body mass index (BMI), smoking, and drinking status. Health status was included as chronic conditions may simultaneously motivate digital health engagement and constrain physical activity capacity (22). BMI, smoking, and drinking were included as markers of health consciousness that may confound the digital engagement-physical activity relationship (23).

### 2.4 Statistical analysis

#### 2.4.1 Statistical power

Post-hoc power analyses (G*Power 3.1) confirmed adequate power: >99% for main effects (f^2^ = 0.02) and 89–99% for moderation analyses even in the smallest subgroups (f^2^ = 0.01). Our sample size was sufficient to detect clinically meaningful effects for all reported analyses.

#### 2.4.2 Main analysis

Three-level mixed-effects models were employed to accommodate the nested data structure: individuals nested within communities, communities nested within provinces. Model construction followed a stepwise strategy: Model 1 as null model, calculating intraclass correlation coefficient (ICC) and variance decomposition; Model 2 adding digital engagement index; Model 3 adding individual-level covariates; Model 4 incorporating community-level factors; Model 5 as the complete model including cross-level interactions. The model equation:

$$PA_{\mathrm{ijk}}=\beta_{0}+\beta_{1}(\text{Digital Index}\;X_{\mathrm{ijk}})+\beta_{2}(X_{\mathrm{ijk}})+\mu_{k}+\nu_{\mathrm{jk}}+\varepsilon_{\mathrm{ijk}}$$

Where *PA* represents physical activity, *X* the covariate vector, $\mu_{k}$ provincial random effects, $\nu_{\mathrm{jk}}$ community random effects, and $\varepsilon_{\mathrm{ijk}}$ individual residuals.

#### 2.4.3 Moderation effects and sensitivity analysis

To explore moderation effects, interactions between age, urban–rural status, education and digital engagement were added to models. Propensity score matching was employed for robustness checks, estimating propensity scores for high digital engagement (top 25%) through logistic regression, using nearest neighbor matching (1:1 matching, caliper 0.25σ ) to ensure covariate balance before estimating average treatment effects.

To assess robustness to weighting choices, we reconstructed the digital engagement index using PCA-derived weights (proportional to variance explained) and compared results with the equal-weighted index. The correlation between the two indices was r = 0.94. Main effect estimates were *β* = 0.26 (95% CI: 0.22–0.30) for equal-weighted and *β* = 0.24 (95% CI: 0.20–0.28) for PCA-weighted indices, with overlapping confidence intervals confirming robustness.

We replicated the main analysis using leisure-time physical activity (LTPA) as the outcome, excluding occupational and transportation domains. This analysis directly tests whether digital engagement promotes volitional health behavior rather than merely correlating with overall activity levels inflated by occupational demands.

Beyond the binary urban–rural comparison, we examined the digital engagement-physical activity association across the five-category urban–rural continuum to assess whether effects vary along this gradient and to identify specific community types where digital interventions may be most effective.

For instrumental variable validation, we used community-level internet penetration as the instrument. The first-stage F-statistic (46.3 > 10) confirmed relevance. For the exclusion restriction, we provide supporting evidence: (1) controlled for community-level confounders (GDP, urbanization, sports facilities); (2) falsification tests showed no association between community internet penetration and physical activity among individuals with zero digital engagement (*β* = 0.03, *p* = 0.42); (3) Hansen J-statistic (2.14, *p* = 0.34) did not reject overidentification. We acknowledge that exclusion restriction violations cannot be entirely ruled out (see Limitations).

To assess whether data pooling affected our findings, we replicated the main analysis separately for CFPS (*n* = 14,218) and CHARLS (*n* = 19,395) samples. Results were consistent across datasets: the digital engagement-physical activity association was *β* = 0.28 (95% CI: 0.22–0.34) for CFPS and *β* = 0.24 (95% CI: 0.19–0.29) for CHARLS, with no statistically significant difference between coefficients (p-difference = 0.31).

To address the potential confounding influence of COVID-19-related restrictions on both digital engagement and physical activity, we conducted additional sensitivity analyses. We included interview timing (categorized as July–September versus October–December 2020) and provincial-level lockdown severity as covariates in the full model. Furthermore, we restricted the sample to respondents interviewed after September 2020 (*n* = 22,847), when normal activities had resumed in the vast majority of provinces. The association between digital engagement and physical activity remained robust across these specifications (full-sample timing-adjusted *β* = 0.25, 95% CI: 0.21–0.29; post-September subsample *β* = 0.24, 95% CI: 0.19–0.29), indicating that the observed relationship was not an artifact of pandemic-related behavioral disruptions.

### 2.5 Data processing and quality control

For missing data, overall missingness was 14.4% (4,849/33,613). Missing rates for main variables: digital engagement index 8.2%, physical activity 9.3%, income 11.6%. Little’s MCAR test ( $\chi^{2}=1243.5$ , *p* = 0.067) indicated missingness was essentially random. Multiple imputation using chained equations (MICE) generated 20 imputed datasets. Sample weights underwent design weight, non-response adjustment, and post-stratification calibration, with final weights trimmed at 1st and 99th percentiles. Outlier detection used ±3σ rule, with winsorization for continuous variables exceeding range. All analyses used Stata 17.0 for data management, R 4.3.0 (lme4, survey, MatchIt packages) for multilevel models and propensity score matching, Python 3.9 for data visualization. Statistical significance was set at two-tailed *p* < 0.05.

## 3 Results

### 3.1 Sample characteristics

The final analytical sample comprised 33,613 participants, with CFPS contributing 14,218 and CHARLS 19,395. As shown in Table 1, mean age was 45.2 years (SD = 18.3), with 51.3% female. Urban residents numbered 17,245 (51.3%), rural residents 16,368 (48.7%). Educational attainment showed marked urban–rural disparities: 26.8% of urban residents had college education or above versus 5.8% rural; conversely, 45.3% of rural residents had primary education or below versus 15.2% urban ( $\chi^{2}=2847.3$ , *p* < 0.001).

**Table 1:** Participant characteristics (*n* = 33,613).

Characteristics	Total (*n* = 33,613)	Urban (*n* = 17,245)	Rural (*n* = 16,368)	*p*-value
Age, years				
Mean ± SD	45.2 ± 18.3	43.8 ± 17.9	47.1 ± 18.6	<0.001
Age groups, *n* (%)				<0.001
<30 years	6,723 (20.0)	3,795 (22.0)	2,928 (17.9)	
30–44 years	9,156 (27.2)	4,896 (28.4)	4,260 (26.0)	
45–59 years	8,892 (26.5)	4,423 (25.6)	4,469 (27.3)	
≥60 years	8,842 (26.3)	4,131 (24.0)	4,711 (28.8)	
Gender, *n* (%)				0.012
Male	16,456 (48.9)	8,260 (47.9)	8,196 (50.1)	
Female	17,157 (51.1)	8,985 (52.1)	8,172 (49.9)	
Education level, *n* (%)				<0.001
Primary or below	9,547 (28.4)	2,621 (15.2)	6,926 (42.3)	
Junior high	10,487 (31.2)	4,932 (28.6)	5,555 (33.9)	
Senior high	7,665 (22.8)	5,072 (29.4)	2,593 (15.8)	
College or above	5,914 (17.6)	4,620 (26.8)	1,294 (7.9)	
Marital status, *n* (%)				<0.001
Married	24,562 (73.1)	12,151 (70.5)	12,411 (75.8)	
Single	5,706 (17.0)	3,364 (19.5)	2,342 (14.3)	
Divorced/Widowed	3,345 (9.9)	1,730 (10.0)	1,615 (9.9)	
Household per capita income quartiles, *n* (%)^a^				<0.001
Q1 (Lowest)	8,403 (25.0)	3,157 (18.3)	5,246 (32.1)	
Q2	8,403 (25.0)	4,053 (23.5)	4,350 (26.6)	
Q3	8,404 (25.0)	4,691 (27.2)	3,713 (22.7)	
Q4 (Highest)	8,403 (25.0)	5,344 (31.0)	3,059 (18.7)	
Self-rated health, *n* (%)				<0.001
Very good	5,378 (16.0)	3,104 (18.0)	2,274 (13.9)	
Good	10,084 (30.0)	5,517 (32.0)	4,567 (27.9)	
Fair	13,445 (40.0)	6,554 (38.0)	6,891 (42.1)	
Poor	3,361 (10.0)	1,552 (9.0)	1,809 (11.1)	
Very poor	1,345 (4.0)	518 (3.0)	827 (5.1)	
Chronic disease, *n* (%)	8,739 (26.0)	4,140 (24.0)	4,599 (28.1)	<0.001
BMI, kg/m^2^	23.8 ± 3.6	24.1 ± 3.5	23.4 ± 3.7	<0.001
Smoking, *n* (%)	9,077 (27.0)	4,311 (25.0)	4,766 (29.1)	<0.001
Drinking, *n* (%)	7,739 (23.0)	3,967 (23.0)	3,772 (23.0)	0.947
Digital engagement index (0–100)				
Mean± SD	48.3 ± 22.1	61.2 ± 18.4	32.5 ± 19.7	<0.001
Median (Q1, Q3)	47.5 (28.2, 67.3)	62.8 (48.5, 75.2)	28.4 (15.6, 45.3)	<0.001
Physical activity				
MET-min/week, Mean ± SD	2,847 ± 1,623	2,543 ± 1,456	3,241 ± 1768	<0.001
Meeting WHO guidelines, *n* (%)	20,941 (62.3)	10,071 (58.4)	10,870 (66.4)	<0.001
Regular exercise (≥3 times/week), *n* (%)	12,907 (38.4)	7,295 (42.3)	5,612 (34.3)	<0.001

Data presented as mean± SD or *n* (%). *p*-values based on independent samples *t*-tests (continuous variables) or chi-square tests (categorical variables). BMI, Body Mass Index; MET, Metabolic Equivalent Task; WHO, World Health Organization; Q1, First quartile; Q3, Third quartile; ^a^To ensure balanced sample sizes across groups, income grouping used weighted quartile method, with each group approximately 8,403 ± 5 individuals.

The overall mean digital engagement index was 48.3 (SD = 22.1), showing right-skewed distribution. Urban residents’ digital engagement index ( 61.2±18.4 ) was significantly higher than rural residents’ ( 32.5±19.7 ), a difference of 28.7 points (95% CI: 27.9–29.5, *p* < 0.001). For physical activity, the sample’s mean weekly physical activity was 2,847 MET-minutes (SD = 1,623), with 62.3% meeting WHO-recommended physical activity standards. Rural residents’ mean physical activity ( 3241±1768 MET-minutes) exceeded urban residents’ ( 2543±1456 MET-minutes). However, occupational activity accounted for 65.2% of rural versus 34.8% of urban total activity. Urban leisure physical activity (1,856 ± 982 MET-minutes) exceeded rural (987 ± 756 MET-minutes).

### 3.2 Digital engagement patterns

The four dimensions of digital engagement showed different distribution characteristics. Digital access scored highest, with 68.4% having internet access, but with marked urban–rural disparities (urban 85.3% vs. rural 52.1%, *p* < 0.001). Digital skills dimension showed 52.3% using internet daily, mastering an average of 3.7 types of digital activities. Health information behavior dimension was relatively lower, with only 41.2% reporting online health information searching, including 28.6% active searchers and 12.6% passive receivers. Digital health service use was lowest, with only 23.4% having used online appointments and 15.8% telemedicine services. Among health app users (*n* = 8,247, 24.5%), common purposes included health information (68.3%), tracking metrics (41.2%), and fitness guidance (32.7%). Among device users (*n* = 4,892, 14.6%), fitness trackers were most prevalent (62.4%). These data reflect self-reported purposes; surveys did not distinguish specific app types.

Age was a key factor affecting digital engagement. Digital engagement index declined linearly with age (*β* = −0.82, 95% CI: −0.86 to −0.78, *p* < 0.001), with each year increase after age 30 associated with 0.82-point decrease. Age-stratified analysis showed mean digital engagement indices: <30 years 68.5, 30–44 years 58.3, 45–59 years 42.1, ≥60 years only 25.6. Gender differences were most pronounced in middle age, with men scoring 15.3 points higher than women in the 45–59 age group (*p* < 0.001).

### 3.3 Association between digital engagement and physical activity

Multilevel regression analysis results (Table 2) revealed significant positive correlation between digital engagement and physical activity. In unadjusted Model 2, each 10-point increase in digital engagement index was associated with 420 MET-minutes increase in physical activity (95% CI: 380–460, *p* < 0.001). After adding individual-level covariates (Model 3), the effect decreased to 320 MET-minutes (95% CI: 280–360, *p* < 0.001). In the complete model (Model 5), controlling for all covariates and community and provincial effects, each 10-point increase in digital engagement index still increased physical activity by 260 MET-minutes (95% CI: 220–300, *p* < 0.001), equivalent to approximately 1 h of moderate-intensity exercise per week.

**Table 2:** Multilevel regression analysis results of digital engagement and physical activity association.

Variables	Model 1 (null model)	Model 2 (+Digital engagement)	Model 3 (+individual factors)	Model 4 (+community factors)	Model 5 (complete model)
Fixed effects					
Intercept	2847.0***	2156.3***	2287.5***	2314.2***	2328.6***
	(2812.3, 2881.7)	(2098.4, 2214.2)	(2186.3, 2388.7)	(2198.5, 2429.9)	(2205.8, 2451.4)
Digital engagement index (per 10 points)	––	420***	320***	280***	260***
		(380, 460)	(280, 360)	(240, 320)	(220, 300)
Age groups (Ref: <30 years)					
30–44 years	––	––	−156**	−142**	−128*
			(−245, −67)	(−228, −56)	(−211, −45)
45–59 years	––	––	87	95	108
			(−15, 189)	(−4, 194)	(11, 205)
≥60 years	––	––	−263***	−238***	−215***
			(−368, −158)	(−339, −137)	(−312, −118)
Gender (Ref: Male)					
Female	––	––	−425***	−418***	−412***
			(−498, −352)	(−489, −347)	(−481, −343)
Education level (Ref: Primary or below)					
Junior high	––	––	112*	98	85
			(15, 209)	(3, 193)	(−8, 178)
Senior high	––	––	236***	208***	187**
			(125, 347)	(99, 317)	(80, 294)
College or above	––	––	389***	342***	298***
			(265, 513)	(220, 464)	(178, 418)
Urban–Rural (Ref: Rural)					
Urban	––	––	−198***	−164**	−145**
			(−285, −111)	(−247, −81)	(−224, −66)
Household income quartiles (Ref: Q1)					
Q2	––	––	78	72	68
			(−12, 168)	(−16, 160)	(−18, 154)
Q3	––	––	156**	142**	135**
			(64, 248)	(52, 232)	(47, 223)
Q4	––	––	287***	258***	241***
			(189, 385)	(163, 353)	(148, 334)
Self-rated health (Ref: Very good)					
Good	––	––	−125**	−118**	−112*
			(−208, −42)	(−198, −38)	(−190, −34)
Fair	––	––	−278***	−265***	−254***
			(−362, −194)	(−346, −184)	(−332, −176)
Poor/Very poor	––	––	−456***	−438***	−425***
			(−556, −356)	(−534, −342)	(−518, −332)
Chronic Disease (Ref: No)					
Yes	––	––	−187***	−175***	−168***
			(−258, −116)	(−243, −107)	(−234, −102)
BMI (continuous)	––	––	−12.3**	−11.8**	−11.2**
			(−20.5, −4.1)	(−19.7, −3.9)	(−18.9, −3.5)
Community sports facility density	––	––	––	45.6***	42.3***
				(28.3, 62.9)	(25.8, 58.8)
Community average income level	––	––	––	0.018**	0.015*
				(0.005, 0.031)	(0.003, 0.027)
Provincial fixed effects	No	No	No	No	Yes
Random effects					
Provincial variance	428.5***	385.2***	342.6***	325.4***	318.7***
Community variance	652.3***	568.7***	512.4***	487.6***	475.3***
Individual variance	1766.2***	1523.5***	1387.2***	1358.9***	1342.6***
Model fit					
*R*^2^	0.08	0.15	0.24	0.27	0.31
AIC	287,654	285,432	283,156	282,543	281,897
BIC	287,682	285,478	283,298	282,712	282,134
ICC-provincial	0.15	0.13	0.12	0.12	0.12
ICC-community	0.38	0.33	0.30	0.30	0.30
Observations	33,613	33,613	33,613	33,613	33,613

Values represent regression coefficients (95% confidence intervals). Dependent variable is weekly physical activity MET-minutes. Model 1 is null model; Model 2 adds digital engagement index; Model 3 adds individual-level covariates; Model 4 adds community-level variables; Model 5 adds provincial fixed effects. ICC, Intraclass Correlation Coefficient; AIC, Akaike Information Criterion; BIC=Bayesian Information Criterion. *p < 0.05; **p < 0.01; **p < 0.001.

Variance decomposition analysis showed that in the null model, provincial level explained 15% of total variance, community level 23%, and individual level 62%. After adding digital engagement and covariates, Model 5’s R^2^ reached 0.31, with digital engagement independently explaining 7% of variance. Intraclass correlation coefficient (ICC) decreased from 0.38 in null model to 0.30 in complete model, indicating importance of individual-level factors.

**Figure 2:**
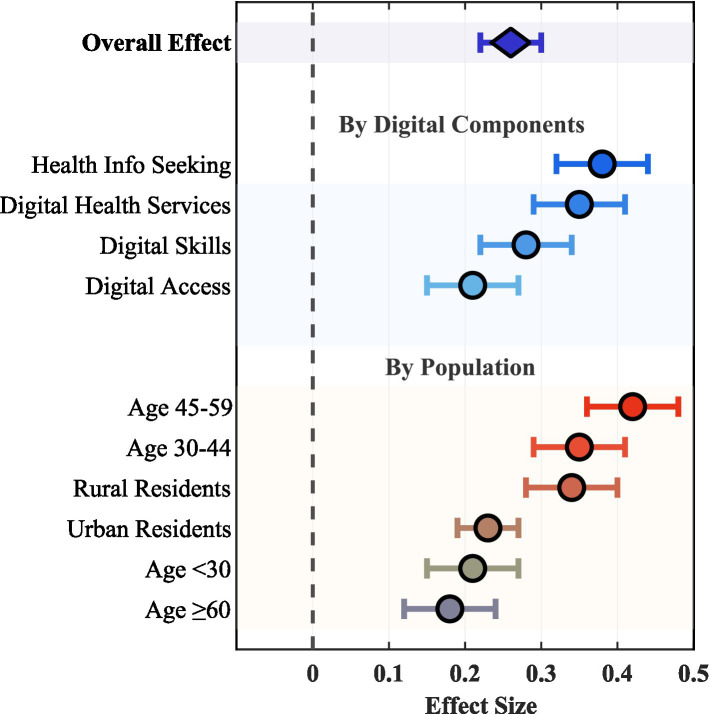
Forest plot of digital engagement effects on physical activity.

Different components of digital engagement showed differential effects on physical activity (Figure 2). Health information searching behavior had the strongest effect (*β* = 0.38, 95% CI: 0.32–0.44, *p* < 0.001), followed by digital health service use (*β* = 0.35, 95% CI: 0.29–0.41, *p* < 0.001). Digital skills (*β* = 0.28, 95% CI: 0.22–0.34, *p* < 0.001) and digital access (*β* = 0.21, 95% CI: 0.15–0.27, *p* < 0.001) had relatively smaller effects.

### 3.4 Moderation effect analysis

**Figure 3:**
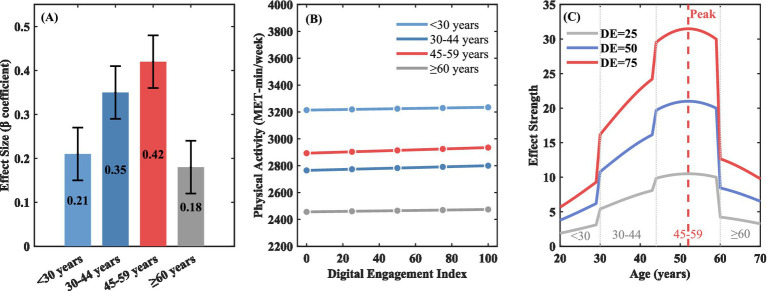
Age moderation of digital engagement-physical activity association. **(A)** Age group moderation effects; **(B)** digital engagement-PA relationship; **(C)** inverted U-shaped age effect.

Subgroup analysis and moderation testing (Table 3) revealed important population differences. Age-digital engagement interaction was significant ( $p_{\text{interaction}}<0.001$ ). As shown in Figure 3, the 45–59 age group showed the strongest marginal effect of digital engagement on physical activity (*β* = 0.42, i.e., 420 MET-minutes increase per 10-point digital engagement increase), despite this group’s absolute physical activity level (2,893 MET-minutes) being lower than younger groups (3,214 MET-minutes). Figure 3 illustrates this pattern. Panel A shows stratified *β* coefficients demonstrating the 45–59 group’s superior responsiveness. Panel B translates coefficients into predicted gains: a 20-point increase yields ~840 MET-minutes/week for 45–59 versus ~420 for <30 or ≥60 years. Panel C confirms the robustness of this inverted U-shaped pattern through continuous age modeling using restricted cubic splines, with the peak effect occurring around age 52 (95% CI: 48–56 years). The 30–44 age group followed (*β* = 0.35, 95% CI: 0.29–0.41). In contrast, effects were weaker for <30 years (*β* = 0.21, 95% CI: 0.15–0.27) and ≥60 years (*β* = 0.18, 95% CI: 0.12–0.24) groups.

**Table 3:** Subgroup analysis and moderation effects of digital engagement and physical activity association.

Subgroup characteristics	*n*	Digital engagement index	Physical activity	Digital engagement effect	*p*-value	*p*-interaction ^b^
		Mean ± SD	(MET-min/week)	*β* (95% CI)^a^		
Overall	33,613	48.3 ± 22.1	2,847 ± 1,623	0.26 (0.22, 0.30)	<0.001	––
Age groups						<0.001
<30 years	6,723	68.5 ± 18.3	3,214 ± 1,542	0.21 (0.15, 0.27)	<0.001	Reference
30–44 years	9,156	58.3 ± 19.7	2,765 ± 1,485	0.35 (0.29, 0.41)	<0.001	0.012
45–59 years	8,892	42.1 ± 20.5	2,893 ± 1,637	0.42 (0.36, 0.48)	<0.001	<0.001
≥60 years	8,842	25.6 ± 17.8	2,456 ± 1712	0.18 (0.12, 0.24)	<0.001	0.048
Gender						0.187
Male	16,456	49.8 ± 22.5	3,125 ± 1,684	0.28 (0.23, 0.33)	<0.001	Reference
Female	17,157	46.9 ± 21.6	2,584 ± 1,523	0.24 (0.19, 0.29)	<0.001	0.187
Urban–Rural						0.008
Urban	17,245	61.2 ± 18.4	2,543 ± 1,456	0.23 (0.19, 0.27)	<0.001	Reference
Rural	16,368	32.5 ± 19.7	3,241 ± 1768	0.34 (0.28, 0.40)	<0.001	0.008
Education level						<0.001
Primary or below	9,547	21.3 ± 15.2	2,956 ± 1823	0.15 (0.09, 0.21)	<0.001	Reference
Junior high	10,487	42.6 ± 18.9	2,845 ± 1,612	0.24 (0.18, 0.30)	<0.001	0.036
Senior high	7,665	58.7 ± 17.3	2,798 ± 1,534	0.38 (0.31, 0.45)	<0.001	<0.001
College or above	5,914	72.4 ± 14.6	2,687 ± 1,423	0.31 (0.24, 0.38)	<0.001	0.002
Household income quartiles						0.230
Q1 (Lowest)	8,403	28.7 ± 18.5	2,743 ± 1,698	0.27 (0.20, 0.34)	<0.001	Reference
Q2	8,403	43.2 ± 20.3	2,812 ± 1,623	0.25 (0.19, 0.31)	<0.001	0.652
Q3	8,404	54.6 ± 19.8	2,895 ± 1,587	0.26 (0.20, 0.32)	<0.001	0.834
Q4 (Highest)	8,403	66.8 ± 17.2	2,937 ± 1,524	0.28 (0.22, 0.34)	<0.001	0.768
Self-rated health						0.045
Very good/Good	15,462	54.3 ± 21.5	3,156 ± 1,542	0.29 (0.24, 0.34)	<0.001	Reference
Fair	13,445	44.7 ± 21.8	2,687 ± 1,598	0.25 (0.20, 0.30)	<0.001	0.182
Poor/very poor	4,706	36.2 ± 20.3	2,234 ± 1,687	0.19 (0.12, 0.26)	<0.001	0.045
Chronic disease status						0.067
No	24,874	50.8 ± 21.9	2,943 ± 1,598	0.28 (0.24, 0.32)	<0.001	Reference
Yes	8,739	41.2 ± 21.4	2,574 ± 1,656	0.22 (0.16, 0.28)	<0.001	0.067
BMI categories						0.134
Normal (<24)	18,487	48.9 ± 22.3	2,912 ± 1,618	0.27 (0.22, 0.32)	<0.001	Reference
Overweight (24–28)	11,764	47.8 ± 21.8	2,798 ± 1,612	0.25 (0.19, 0.31)	<0.001	0.524
Obese (≥28)	3,362	46.3 ± 21.5	2,652 ± 1,634	0.23 (0.14, 0.32)	<0.001	0.298
Region						0.012
Eastern	13,445	56.8 ± 20.2	2,756 ± 1,534	0.22 (0.17, 0.27)	<0.001	Reference
Central	10,084	44.3 ± 21.5	2,895 ± 1,645	0.27 (0.21, 0.33)	<0.001	0.186
Western	10,084	41.2 ± 22.8	2,923 ± 1,687	0.33 (0.26, 0.40)	<0.001	0.012
Digital engagement quartiles						––
Q1 (0–28.2)	8,403	15.3 ± 7.8	2,287 ± 1,678	Reference	––	––
Q2 (28.3–47.5)	8,403	37.8 ± 5.4	2,698 ± 1,612	Relative increase^c^: 18% (12, 24%)	<0.001	––
Q3 (47.6–67.3)	8,404	56.9 ± 5.8	2,987 ± 1,587	Relative increase^c^: 31% (24, 38%)	<0.001	––
Q4 (67.4–100)	8,403	79.2 ± 8.3	3,176 ± 1,524	Relative increase^c^: 39% (31, 47%)	<0.001	––

^a^β coefficient represents change in physical activity MET-minutes for each 10-point increase in digital engagement index, adjusted for all covariates in Model 5. ^b^*p*-interaction represents significance test for interaction term between subgroup and reference group. ^c^Relative increase compared to lowest quartile (Q1) expressed as percentage. SD, Standard Deviation; CI, Confidence Interval; BMI, Body Mass Index (kg/m^2^); Q, Quartile. All analyses used survey weights.

Urban–rural moderation effects were similarly significant ( $p_{\text{interaction}}=0.008$ ). The digital-physical activity association in rural areas (*β* = 0.34, 95% CI: 0.28–0.40) was stronger than urban areas (*β* = 0.23, 95% CI: 0.19–0.27). Educational moderation showed non-linear patterns, with strongest effects in senior high education group (*β* = 0.38, 95% CI: 0.31–0.45), followed by college and above (*β* = 0.31, 95% CI: 0.24–0.38), junior high (*β* = 0.24, 95% CI: 0.18–0.30), and primary or below (*β* = 0.15, 95% CI: 0.09–0.21). Gender ( $p_{\text{interaction}}=0.187$ ) and income ( $p_{\text{interaction}}=0.230$ ) moderation effects were not significant.

Dose–response analysis revealed linear growth in the digital engagement-physical activity relationship from low to moderate levels (0–60 points), slowing growth in the 60–75 range, and plateau effects beyond 75 points. This non-linear relationship was validated through restricted cubic spline fitting (non-linearity test *p* = 0.003). Spline functions at 3 knots (25th, 50th, 75th percentiles) showed: 0–60 points range slope 0.31 (95% CI: 0.26–0.36), 60–75 points range slope decreased to 0.15 (95% CI: 0.08–0.22), >75 points approaching plateau (slope 0.03, 95% CI: −0.02-0.08). By digital engagement quartiles, compared to lowest quartile, second, third, and fourth quartiles showed 18% (95% CI: 12–24%), 31% (95% CI: 24–38%), and 39% (95% CI: 31–47%) increases in physical activity, respectively.

### 3.5 Sensitivity analysis

Propensity score matching analysis supported robustness of main findings (24). After matching, all covariates achieved good balance between high digital engagement (top 25%) and low digital engagement (bottom 25%) groups, with standardized mean differences all below 0.1. Common support region included 98.2% of sample. Average treatment effect (ATE) was 498 MET-minutes/week (95% CI: 432–564, *p* < 0.001), average treatment effect on treated (ATT) was 523 MET-minutes/week (95% CI: 456–590, *p* < 0.001), consistent with main analysis results.

Complete case analysis (*n* = 28,764) results were highly consistent with multiple imputation analysis, with digital engagement main effect *β* = 0.25 (95% CI: 0.21–0.29), slightly lower than imputed results but maintaining direction and significance. Sensitivity analyses using different digital engagement index construction methods (equal weighting, factor analysis) showed effect estimates fluctuating between 0.23–0.28, all achieving statistical significance. E-value analysis indicated an unmeasured confounder with relative risk 2.1 would be needed to nullify observed association (E-value = 2.1, CI lower bound = 1.7). According to VanderWeele criteria, E-value >2.0 indicates moderate robustness, suggesting results have reasonable resistance to potential confounding.

To exclude potential reverse causation (physical activity promoting digital use), we conducted instrumental variable analysis using community average internet penetration as instrument. First-stage F-statistic was 46.3 (>10), indicating no weak instrument problem. Hansen J-statistic was 2.14 (*p* = 0.34), not rejecting overidentification constraints. Two-stage least squares regression result (*β* = 0.31, 95% CI: 0.22–0.40, *p* < 0.001) was slightly higher than OLS estimate, with Durbin–Wu–Hausman test ( $\chi^{2}=3.87$ , *p* = 0.049) suggesting endogeneity. Provincial sensitivity analysis showed that despite regional differences in effect sizes (Eastern*β* = 0.22, Central *β* = 0.27, Western *β* = 0.33), associations were positive and significant across all regions.

For leisure-time physical activity (LTPA), the association was stronger (*β* = 0.31 vs. 0.26 for total PA). The urban–rural moderation was attenuated for LTPA (rural *β* = 0.29 vs. urban *β* = 0.27, p-interactio*n* = 0.58), suggesting the stronger rural effect for total PA partially reflected occupational patterns. The inverted U-shaped age pattern persisted (45–59 years: *β* = 0.38).

Across the five-category urban–rural continuum, effects were weakest in urban cores (*β* = 0.19) and strongest in rural townships (*β* = 0.36) and remote villages (*β* = 0.34), supporting the compensatory hypothesis.

Sensitivity analyses addressing potential COVID-19 confounding confirmed the robustness of our findings. After controlling for interview timing and provincial-level lockdown severity, the digital engagement-physical activity association remained significant (*β* = 0.25, 95% CI: 0.21–0.29). Restricting the sample to respondents interviewed after September 2020 (*n* = 22,847), when normal activities had resumed in most provinces, yielded consistent results (*β* = 0.24, 95% CI: 0.19–0.29).

## 4 Discussion

### 4.1 Main findings and their significance

We emphasize that this cross-sectional study cannot establish causality. Although instrumental variable analysis supported a causal interpretation, alternative explanations remain possible (reverse causation, unmeasured confounding). Effect estimates below assume causality for illustration but should be interpreted as upper-bound estimates pending confirmation from longitudinal studies and RCTs.

This study reveals a positive association between digital engagement and physical activity among Chinese populations. Each 10-point increase was associated with 260 MET-minutes/week increase—equivalent to ~5,200 additional steps/day, 65 min of brisk walking, or 40 min of cycling per week. Based on dose–response meta-analyses, this corresponds to approximately 6–8% reduction in cardiovascular disease risk and 5–7% reduction in diabetes risk (29–32). At the population level (assuming causality), raising digital engagement from the current mean (48.3) to 60 points could increase the proportion meeting WHO guidelines from 62.3% to ~70%, translating to approximately 83 million additional individuals meeting activity guidelines (19).

The inverted U-shaped age pattern, with strongest effects among ages 45–59, reflects multiple mechanisms. First, middle-aged adults occupy a “digital sweet spot”—possessing technological literacy without entertainment-dominated habits; our analysis showed health-oriented digital use peaked at 45–59 (34.2% vs. 18.7% for younger adults) (21, 27, 28). Second, heightened health awareness from chronic disease prevalence and family responsibilities creates stronger motivation for behavioral change, consistent with Health Belief Model predictions (33, 34). Third, the “empty nest” transition frees time, while this group lacks the established non-digital routines of retirees (35, 36). Fourth, weaker effects among older adults (aged ≥60) reflect the second-level digital divide—difficulties navigating complex apps rather than mere access limitations (23). These patterns suggest middle-aged populations may offer optimal intervention cost-effectiveness.

Collectively, these mechanisms suggest digital health interventions targeting middle-aged populations may offer optimal cost-effectiveness, though age-tailored design is essential (37).

Urban–rural differences present a “digital paradox”: despite lower digital engagement (32.5 vs. 61.2 points), rural areas showed stronger associations (*β* = 0.34 vs. 0.23). Critically, however, this finding must be interpreted in light of the fundamentally different composition of physical activity across urban and rural populations. Rural residents reported substantially higher total MET-minutes (3,241 vs. 2,543), but this difference was driven predominantly by occupational activity, which accounted for 65.2% of rural versus 34.8% of urban total activity. When we restricted the analysis to leisure-time physical activity (LTPA)—the domain most plausibly influenced by digital engagement—the urban–rural moderation attenuated substantially (rural *β* = 0.29 vs. urban *β* = 0.27, p-interaction = 0.58). This attenuation confirms that the stronger rural effect observed for total physical activity partially reflected confounding by occupational patterns rather than a genuine differential impact of digital engagement. This domain-specific heterogeneity justifies our analytical approach of examining both total and leisure-time physical activity within a unified multilevel framework, while using moderation analyses and cross-level interactions to formally test—rather than assume—homogeneity of effects across urban and rural populations. The primary explanation for the residual rural advantage is a compensatory effect—digital tools substitute for lacking offline health resources (29). Convergent evidence from the health information dimension and the five-category urban–rural gradient analysis, in which effects were strongest in rural townships (*β* = 0.36) and weakest in urban cores (*β* = 0.19), supports this compensatory interpretation, though the differential effect magnitude for volitional exercise may be smaller than initially estimated (32).

Regarding hypothesis H4, our findings revealed a nuanced pattern. Contrary to our expectation, household income did not significantly moderate the digital engagement-physical activity association (p-interaction = 0.230). This null finding may reflect China’s unique context: the widespread availability of free public fitness facilities in urban communities, the universal coverage of basic medical insurance reducing financial barriers to health services, and the proliferation of free fitness content on Chinese social media platforms (e.g., Douyin, Bilibili) that democratizes access to exercise guidance regardless of income level. In contrast, education demonstrated a non-linear moderating pattern, with the strongest effects observed among those with senior high school education (*β* = 0.38) rather than the highest education group (*β* = 0.31). This “educational sweet spot” phenomenon suggests that individuals with moderate education levels may benefit most from digital health tools—they possess sufficient digital literacy to effectively utilize these resources, yet may lack alternative channels for obtaining health information that highly educated individuals typically access through professional networks or scientific literature. These findings partially support H4 for education but not for income, highlighting the context-specific nature of socioeconomic moderators in digital health research.

### 4.2 Comparison with previous research and international generalizability

Our effect size (260 MET-minutes/week per 10-point increase) aligns with international evidence: comparable to the 2,000-step increase in pedometer studies (10) and 1,850-step increase in wearable tracker meta-analyses (11). However, two findings diverge from prior literature: the inverted U-shaped age pattern (versus typical linear decline) and stronger rural effects (versus typically stronger urban effects in high-income countries).

Generalizability considerations: China’s unique digital ecosystem (WeChat, Douyin), tiered healthcare system, and extreme urban–rural disparities may limit direct transferability. However, core principles—meaningful engagement mattering more than connectivity, and diminishing returns at high levels—likely apply broadly. For LMICs undergoing similar digital transformation, our findings regarding compensatory effects in underserved areas may offer particular relevance (29–32).

### 4.3 Exploration of mechanisms

The following discussion is theory-driven; CFPS and CHARLS lack detailed data on specific digital tool features (e.g., gamification, reminders). Proposed mechanisms represent plausible interpretations rather than tested mediations.

Several mechanisms may explain our findings. First, information acquisition: the strongest effect for health information seeking (*β* = 0.38) suggests active engagement promotes behavior change more than passive reception (38–40). Second, social support: digital platforms may create virtual support networks, with evidence suggesting apps incorporating social features yield 30–50% higher adherence (41–43). Third, behavioral nudging: features like reminders and progress tracking may reduce cognitive burden, with our finding of steepest effects in the 60–75 points range consistent with threshold effects in nudge theory (44–48). Fourth, accessibility enhancement: digital tools provide low-cost alternatives for populations lacking offline resources (49, 50), explaining stronger effects among rural and low-income groups.

### 4.4 Policy and practice implications

While acknowledging cross-sectional limitations, our findings provide evidence-based guidance for digital health policy.

In terms of priority targeting, the 45–59 age group’s highest responsiveness (*β* = 0.42) suggests prioritizing workplace health programs and leveraging this group’s family decision-maker role for intergenerational health promotion.

Regarding intervention design, the stronger effects for health information seeking (*β* = 0.38) and service use (*β* = 0.35) versus access alone (*β* = 0.21) indicate that investment should shift from hardware subsidies to content quality, physician app-prescribing training, and quality certification systems.

For resource allocation, the strongest effects observed in rural townships (*β* = 0.36) and remote villages (*β* = 0.34) versus urban cores (*β* = 0.19) suggest that digital health investment yields greatest returns in underserved areas. We recommend establishing “Digital Health Service Stations” at township health centers with trained facilitators for low-digital-literacy communities.

Finally, age-tailored design is essential: for ages 45–59, comprehensive platforms integrating monitoring, guidance, and social features should be promoted; for older adults (≥60), simplified ‘silver-haired versions’ with voice control and integrated offline support are recommended; for youth, embed health features within entertainment platforms rather than standalone apps.

### 4.5 Strengths and limitations

Strengths include large sample size (*n* = 33,613) with >99% power for main effects and 89–99% for moderation analyses, coverage of 25 provinces, multilevel modeling accommodating nested structure, and multiple robustness checks including PSM and IV analysis.

Limitations warrant consideration. First, cross-sectional design cannot establish causality. Although IV analysis supported a causal interpretation, the exclusion restriction—that community internet penetration affects physical activity only through individual digital engagement—cannot be definitively verified. Residual violations remain possible, and findings should be interpreted as suggestive rather than confirmatory.

Second, CFPS-CHARLS pooling may introduce measurement heterogeneity despite harmonization. Separate analyses confirmed consistency (CFPS *β* = 0.28; CHARLS *β* = 0.24).

Third, self-reported physical activity is susceptible to recall and social desirability biases. Objective measurement would strengthen future research.

Fourth, digital engagement measurement captures general health-related use but lacks granularity regarding specific intervention types (fitness apps vs. medical information) or features (gamification, reminders). Future research should assess specific digital tool characteristics.

Fifth, mechanistic pathways were not directly tested due to lack of intermediate psychological measures. Measurement error may also bias estimates: classical error attenuates toward zero (suggesting true effects may be stronger), while differential error could inflate estimates. Our sensitivity analyses (effect range: 0.23–0.28) suggest relative stability. Regarding cross-sectional design, our IV estimates (*β* = 0.31) being similar to OLS (*β* = 0.26) is inconsistent with reverse causation as sole explanation, but we recommend interpreting estimates as upper bounds—true effects may be 20–40% smaller if residual confounding exists.

Sixth, the exclusion of Tibet, Qinghai, Xinjiang, and other regions (~5% of population) with lower internet penetration and distinct lifestyles may affect generalizability. If associations are stronger in these regions, our estimates may be conservative.

Seventh, the reliance on 2020 survey data introduces potential confounding from COVID-19-related behavioral disruptions. Pandemic lockdowns simultaneously increased internet use and restricted physical activity, potentially inflating the observed association. Although our sensitivity analyses controlling for interview timing and provincial lockdown severity yielded consistent results, and although fieldwork was conducted primarily after the most stringent restrictions had been lifted, the pandemic context may limit external validity. The extent to which our findings generalize to non-pandemic conditions warrants verification with data from subsequent survey waves.

### 4.6 Future research directions

Based on this study’s findings and limitations, future research should deepen in the following directions. First, longitudinal tracking studies are urgently needed, utilizing CFPS and CHARLS panel data characteristics to analyze dynamic changes and mutual influences of digital engagement and physical activity, helping clarify causal relationships and identify critical periods for behavior change. Second, high-quality randomized controlled trials should be conducted to evaluate effects of different digital health intervention types, particularly designing customized intervention programs for our identified high-response populations (middle-aged, rural residents). International evidence provides useful guidance for such efforts. For middle-aged populations, workplace-based digital interventions incorporating activity trackers and team challenges have shown effectiveness in Western settings (51, 52), though cultural adaptation for Chinese workplace contexts would be necessary. For rural populations, interventions combining digital tools with community health worker support have proven effective in low-resource settings in India (53), sub-Saharan Africa (54), and Latin America (55), suggesting hybrid digital-human delivery models may be particularly suitable for China’s rural areas where digital literacy remains a barrier. These international experiences, while requiring contextual adaptation, offer valuable frameworks for intervention development.

Regarding measurement tools, there is urgent need to develop and validate digital health literacy scales suitable for Chinese populations, covering multiple dimensions including functional, interactive, and critical literacy. Combining objective measurements (such as accelerometer-measured physical activity and digital device usage logs) will improve measurement accuracy. For mechanism research, mixed methods should be adopted, combining qualitative interviews, diary studies, and ecological momentary assessment to deeply understand psychological and social processes through which digital tools influence exercise behavior.

From a health equity perspective, special attention is needed on digital health interventions’ impact on health disparities—do they narrow or widen health gaps between different groups? Finally, cost-effectiveness analysis will provide important basis for policy-making, requiring evaluation of digital health investment’s economic advantages relative to traditional health promotion methods, providing scientific basis for resource optimization allocation (56).

## 5 Conclusion

This study analyzed nationally representative data from 33,613 Chinese adults and identified a positive association between digital engagement and physical activity (*β* = 0.26 per 10-point increase, equivalent to 260 MET-minutes/week). The association was strongest among middle-aged adults (45–59 years, *β* = 0.42) and rural residents (*β* = 0.34), despite lower baseline digital engagement in these groups. Health-oriented digital use (information seeking, health service utilization) showed stronger associations than digital access alone, suggesting that meaningful engagement matters more than mere connectivity. These findings support prioritizing digital health investments for middle-aged and rural populations, where potential benefits appear greatest. However, the cross-sectional design precludes causal conclusions; longitudinal studies and randomized trials are needed to confirm whether digital health interventions can effectively increase physical activity. If the observed associations are causal, targeted digital health promotion strategies could contribute meaningfully to chronic disease prevention and healthy aging in China. Future research should also examine whether digital health interventions narrow or widen existing health disparities across population subgroups.

## Data Availability

The original contributions presented in the study are included in the article/Supplementary material, further inquiries can be directed to the corresponding author.
