# Supplementary material for: Digital engagement and physical activity patterns across Chinese populations: a secondary analysis of national survey data
Source: Front Public Health. 2026 May 20;14:1726172. doi: 10.3389/fpubh.2026.1726172 (PMC13230098; doi:10.3389/fpubh.2026.1726172)
Supplement: Supplementary file 1 [file Table_1.DOCX]

**Supplementary Materials**

**Digital Engagement and Physical Activity Patterns Across Chinese Populations: A Secondary Analysis of National Survey Data**

**Supplementary Table S1.** Variable Harmonization Crosswalk Between CFPS and CHARLS

| **Variable** | **CFPS 2020** | **CHARLS 2020** | **Harmonization Notes** |
| --- | --- | --- | --- |
| **Dependent Variable** | |  |  |
| Physical activity (vigorous) | qp601, qp602 | da051_1_, da052_1_ | IPAQ protocol; MET=8.0 |
| Physical activity (moderate) | qp603, qp604 | da051_2_, da052_2_ | IPAQ protocol; MET=4.0 |
| Physical activity (walking) | qp605, qp606 | da051_3_, da052_3_ | IPAQ protocol; MET=3.3 |
| **Independent Variable** | |  |  |
| Internet access | qn12001 | da065 | Binary (1=yes, 0=no) |
| Smartphone ownership | qn401 | da066 | Smartphone=1, other=0 |
| Computer ownership | familyasset | hc007 | Extracted from asset list |
| Internet use frequency | qn12002 | da067 | Standardized to 5-point scale |
| Digital activities variety | qn12003-qn12010 | da068_1_ to da068_8_ | Sum of activity types (0-8) |
| Online health info search | qn12011 | da069 | Binary (1=yes, 0=no) |
| Health app use | qn12012 | da070 | Binary (1=yes, 0=no) |
| Online appointment | qn12013 | da071 | Binary (1=yes, 0=no) |
| Telemedicine use | qn12014 | da072 | Binary (1=yes, 0=no) |
| Health monitoring device | qn12015 | da073 | Binary (1=yes, 0=no) |
| **Covariates** |  |  |  |
| Age | cfps_age | r_age | Continuous (years) |
| Gender | cfps_gender | rgender | 1=male, 0=female |
| Education | cfps_edu | bd001 | 4 categories |
| Marital status | qe104 | be001 | 3 categories |
| Household income | fincome1 | htotal | Per capita, 2020 CNY |
| Employment status | qg303 | fa001 | Binary |
| Urban-rural (hukou) | hukou_local | bc001 | Agri=rural, Non-agri=urban |
| Province | provcd | province | 25/28 provinces |
| Self-rated health | qp201 | da001 | 5-point Likert scale |
| Chronic disease | qp501 series | da007 series | Any condition=1 |
| BMI | qp801, qp802 | da021, da022 | Weight(kg)/Height(m)² |
| Smoking status | qp901 | da059 | Current smoker=1 |
| Drinking status | qp1001 | da061 | Current drinker=1 |

*Notes: CFPS = China Family Panel Studies; CHARLS = China Health and Retirement Longitudinal Study. For overlapping age groups (45+ years), CFPS was used as primary source to avoid duplicate sampling. Survey weights were recalibrated using post-stratification to 2020 Census benchmarks.*
